# Stages of Granulomatous Response Against Histozoic Metazoan Parasites in Mullets (Osteichthyes: Mugilidae)

**DOI:** 10.3390/ani11061501

**Published:** 2021-05-21

**Authors:** Marta Polinas, Francesc Padrós, Paolo Merella, Marino Prearo, Marina Antonella Sanna, Fabio Marino, Giovanni Pietro Burrai, Elisabetta Antuofermo

**Affiliations:** 1 Department of Veterinary Medicine, University of Sassari, 07100 Sassari, Italy; paolomerella@uniss.it (P.M.); msanna@uniss.it (M.A.S.); gburrai@uniss.it (G.P.B.); eantuofermo@uniss.it (E.A.); 2 Fish Diseases Diagnostic Service, Facultat de Veterinaria, Universitat Autonoma de Barcelona, 08193 Barcelona, Catalonia, Spain; francesc.padros@uab.cat; 3 Fish Disease Laboratory, State Veterinary Institute of Piedmont, Liguria and Aosta Valley, 10154 Torino, Italy; marino.prearo@izsto.it; 4 Department of Chemical, Biological, Pharmaceutical and Environmental Sciences, University of Messina, 98166 Messina, Italy; fabio.marino@unime.it; 5 Mediterranean Center for Disease Control (MCDC), University of Sassari, 07100 Sassari, Italy

**Keywords:** disease staging, wild fish, immunohistochemistry, protozoan, Trematoda

## Abstract

**Simple Summary:**

Parasitic diseases represent a common issue in fish and, when histozoic forms are present, this elicits a chronic inflammatory reaction leading to granuloma formation. Despite the large knowledge of granuloma formation due to parasites in visceral organs, little is known about the development and the evolutive stages of granulomas in naturally infected fish. Mullets (Osteichthyes: Mugilidae) are a widespread euryhaline fish species that harbor different parasites, thus representing a suitable model for the study of parasite-induced granulomas. Combining histopathology and immunohistochemical tools, we identified three developmental granuloma stages (pre-granuloma, intermediate, and late stage), that ranged from an intact parasite with mild signs of tissue reaction to the formation of a structured granuloma. The identified histological patterns could be reliable tools in the staging of the granulomatous response associated with histozoic parasites and are an attempt to broaden the knowledge of the inflammatory response in different host–parasite systems.

**Abstract:**

Histozoic parasite–fish host interaction is a dynamic process that leads to the formation of a granuloma, a specific chronic inflammatory response with discernible histological features. Mullets (Osteichthyes: Mugilidae) represent a suitable model concerning the development of such lesions in the host–parasite interface. The present work aimed to identify granuloma developmental stages from the early to the late phase of the infection and to characterize the immune cells and non-inflammatory components of the granuloma in different stages. For this purpose, 239 mullets were collected from 4 Sardinian lagoons, and several organs were examined by combining histopathological, bacteriological, and immunohistochemical methods. Granulomas associated with trematode metacercariae and myxozoan parasites were classified into three developmental stages: (1) pre-granuloma stage, characterized by intact encysted parasite and with no or mild tissue reaction; (2) intermediate stage, with partially degenerated parasites, necrosis, and a moderate number of epithelioid cells (ECs); and (3) late stage, with a necrotic core and no detectable parasite with a high number of ECs and fibroblasts. The three-tier staging and the proposed morphological diagnosis make it conceivable that histopathology could be an essential tool to evaluate the granulomas associated with histozoic parasitic infection in fish.

## 1. Introduction

The chronic inflammatory response is a non-specific reaction against a multitude of factors, such as harmful substances, bacteria, mycobacteria, fungi, and parasites, particularly common in fish [1,2,3,4,5,6,7,8,9,10,11,12,13,14]. Chronic inflammation is a complex combination of immune cell action that involves mainly macrophages, epithelioid cells (ECs), fibroblasts, and piscine mast cells (MCs) usually organized as a granulomatous reaction to isolate the pathogens or foreign materials [15,16]. Phagocytosis is a primordial and preserved mechanism of innate immunity in vertebrates, mainly exerted by macrophages, that are considered specialized phagocytes [17,18]. When the noxa overcomes the phagocytic ability of the host, the organism triggers more complex mechanisms to confine the intruder into host tissues, leading to granuloma in affected organs [18]. The size, structure, organization, and composition of these granulomas may present differences according to the causative agent.

In natural and experimental infection due to bacterial and particularly mycobacterial and oomycetes species, granulomas have been described as a core of macrophages and bacteria associated with central necrosis, surrounded by a fibrous layer mostly evident in the later stages of infective granulomas [19,20,21]. Unlike mammals, in fish, the presence of giant cells has been only occasionally described in parasite granuloma [22,23].

It is well known that also some histozoic parasites (e.g., amoebae, myxozoans, larval stages of trematodes, and nematodes) can elicit chronic granulomatous inflammation [24].

More details of the cellular components of granuloma and their distribution have been provided by electron microscopy studies, where ECs have been typically found near or in contact with the parasite body or cyst wall [25]. Although considered transformed macrophages, ECs mainly act to confine the pathogen due to the presence of interdigitation and desmosomes and expression of cytokeratins [2,26,27,28]. Around EC layers, an outer sheet of fibroblasts has been frequently reported, intermingled with collagen fibers, to further reinforce pathogen incarceration and favor tissue remodeling [29,30,31]. Moreover, fibroblasts, considered for a long time as plain structural components, have been recently elevated to crucial effectors in the progression and resolution of chronic inflammation in humans, through crosstalk with mast cells and eosinophils [32]. Furthermore, ultrastructural observation showed the presence of MCs near or in contact with fibroblasts, in an apparent degranulation state [33]. A role of MCs in promoting collagen production has been proposed in teleosts, similarly to what was observed in humans in association with parasitic diseases, where these cells are involved in the pathogenesis of fibrotic processes via the secretion of profibrotic substances [34].

Rodlet cells (RCs), although not a regular component in parasite granulomas, increase in the course of infectious diseases, parasitic infections, and environmental pollutants [35,36].

Granuloma formation is a dynamic process, and many times the structure of these specific chronic inflammatory responses can give useful information about the timing, evolution, and prognosis of the disease.

Nevertheless, detailed studies on granuloma progression against histozoic parasites have not been carried out, although numerous details of parasite granuloma cellular components have been reported both in saltwater and freshwater fish [10,30,37]. Among brackish water fish species, the mullets (Osteichthyes: Mugilidae) are widely investigated in parasitological studies [38,39], so they could represent a suitable model to study different host–parasite interaction systems [40,41]. Moreover, as mullets can harbor trematode parasites with a zoonotic potential (i.e., Heterophyidae), several studies have widely investigated the presence of such parasites also concerning human consumption of raw fish [38,42]. However, only a single study on mullets concerning the lesion development in the host–parasite interface after the infection of *Labratrema minimus* (Trematoda, Bucephalidae) metacercariae has been accomplished [43]. The presence of large resident populations of mullets with already described metazoan parasites in Sardinian lagoons [39] allows a unique opportunity for a detailed study of the complexity and dynamics of the host–parasite relationship.

The present work aims to characterize the histological features of the granulomatous inflammatory response in visceral organs of mullets naturally infected with trematode and myxozoan parasites.

For this purpose, different developmental stages of granuloma, from the early to the late phase of the histozoic parasite infection, were identified using discernible histological features, and afterward, the immune cells and other non-inflammatory components associated with the different stages of the granuloma were characterized by histochemical and immunohistochemical tools.

## 2. Materials and Methods

### 2.1. Sampling

From July to October 2014, 239 mullets were collected by fishing nets from 4 different coastal lagoons in Sardinia (western Mediterranean Sea): 60 from Calich lagoon (40°35′46.1″ N 8°17′59.69″ E; Alghero-SS), 60 from San Teodoro lagoon (40°47′51.71″ N 9°40′00.05″ E; San Teodoro-SS), 60 from Mistras lagoon (39°54′16.09″ N 8°27′21.74″ E; Cabras-OR), and 59 from Marceddì lagoon (39°42′40.01″ N 8°31′06.53″ E; Terralba-OR).

Identification of Mugilidae species revealed a heterogeneous group composed of *Mugil cephalus* (11), *Chelon labrosus* (35), *Chelon auratus* (83), and *Chelon ramada* (110), variably combined in the different lagoons. All the specimens were identified at species level following Ben-Tuvia (1986) and Bauchot (1987); fish names conform to FishBase [44,45,46]. Sampled fish species are reported in Table 1.

### 2.2. Parasitological Analysis

The mullets were transferred to the Department of Veterinary Medicine, University of Sassari, and the internal organs (heart, liver, spleen, and kidney) of a subsample of 12 fish were analyzed for the presence of tissue parasites to identify the main taxa present. Squashes and scrapings of organs were placed on slides, pressed under a coverslip, and observed under an optical microscope. The parasites found were trematode metacercariae (TM) and myxozoan plasmodia (MP), and they were identified to the lowest taxonomic level possible according to the observable characters, following the specialistic literature [42,47,48,49,50,51].

### 2.3. Bacteriological Analysis

A culture test was carried using the anterior kidney of the 239 subjects on a primary isolation medium (blood agar). Colonies present in the plates were cloned and identified by phenotypic (Gram staining, oxidase, growth temperature, different NaCl concentration, pH range), biochemical (API System, bioMérieux), mass spectrometry (MALDI-TOF), and molecular (using generic primers and subsequent sequencing) methods.

Fresh tissues from sampled organs (liver and spleen) were homogenized and decontaminated and inoculated in Stonebrink medium tubes and 2 Löwenstein–Jensen medium tubes (Microbiol, Uta, Cagliari, Italy) for mycobacteria culture. Decontamination of the samples was carried out using 1.5% cetylpyridinium chloride, following the standard protocol aimed at eliminating contaminating bacteria, in order to allow mycobacteria growth on specially sown soils [52].

### 2.4. Histology and Immunohistochemistry

Samples of the liver, spleen, heart, and kidney of the 239 mullets were collected and immediately fixed in 10% buffered formalin for 48 h, dehydrated with increasing alcohol concentrations and xylene in an automatic tissue processor, and paraffin embedded. Sections of 3 μm thickness were obtained with a microtome (RM2245, Leica Biosystems, Wetzlar, Germany) and stained with hematoxylin and eosin in an automatic multistainer (ST5020, Leica Biosystems, Wetzlar, Germany). Slides were then evaluated at light microscopy (Nikon Eclipse 80i, Amsterdam, Netherlands). Lesions found in visceral organs (liver, spleen, heart, and kidney) were evaluated according to the parasite class involved [53,54].

A total of 75 well-preserved granulomatous lesions from 47 of the 239 fish were included in this study. All granulomas were selected considering different organs and different classes of parasites involved. Lesions were classified into three developmental stages based on the evaluation of selected histological features (i.e., state of parasites and necrosis) and were investigated to evaluate different structural components (i.e., epithelioid cells and fibroblasts) by immunohistochemistry (IHC). Serial sections were mounted on positively charged slides (Superfrost, Fisher Scientific) for IHC according to a previously developed protocol [55,56,57]. Briefly, slides were immersed for 20 min in a 98 °C, preheated solution (WCAP, citrate pH 6, BiOptica, Milan, Italy) for antigen unmasking. Tissues were blocked for endogenous peroxidase (Dako REAL Peroxidase-Blocking Solution S2023, Dako, Glostrup, Denmark) and non-specific binding with 2.5% normal horse serum (ImmPRESS reagent kit, Vector Labs, Burlingame, CA, USA). Sections were incubated overnight at 4 °C with the following primary antibodies: anti-cytokeratin AE1–AE3, dilution 1:250 (Dako, Monoclonal mouse Anti-Human Cytokeratin CKAE1/AE3) to evaluate epithelioid cells and anti-Vimentin, dilution 1:500 (Dako, Monoclonal Mouse Anti-Vimentin clone V9) to evaluate fibroblasts. Sections were then incubated with an anti-mouse/rabbit secondary antibody (ImmPRESS reagent kit—peroxidase—MP-7500; Vector Laboratories, Burlingame, CA, USA) for 30 min at room temperature and treated with 3,3′-Diaminobenzidine (DAB) chromogen (ImmPACT DAB; Vector Laboratories). Tissues were then counterstained with hematoxylin, dehydrated, and mounted (Eukitt mounting medium; BiOptica, Milano, Italy). Appropriate positive and negative controls were included for each antibody.

The presence of collagen, mast cells, and rodlet cells was also evaluated with Masson’s trichrome and Giemsa staining, respectively [58]. To investigate the potential presence of mycobacteria within the granuloma, Ziehl-Neelsen staining was performed.

Granulomas were further evaluated for their proximity to the blood vessel and assigned to one of the following three categories: close to the vessel (granuloma within the thickness of the vascular wall), near the vessel (granuloma in contact, less than 20 microns, with the external part of the vascular wall), far from the vessel (granuloma distance from the vessel >100 microns or not present in the field of view at 200× magnification). Additionally, macrophage aggregates (MAs) associated with granulomas were also evaluated. The 75 granulomas were assigned to the “present” or “absent” categories based on the presence or absence of macrophage aggregates near the granuloma in a field of view at 200× magnification.

### 2.5. Image Analysis

Each lesion was microphotographed and images were analyzed using a Java-based open-source image processing program (https://imagej.net/ImageJ, accessed on 15 May 2020). The quantitative evaluation of the collagen area, associated with the lesions, was accomplished using ImageJ software on microphotographs taken at 200× magnification on slides stained with Masson’s trichrome. In detail, the total area of the granuloma was selected by the “freehand selection” tool, measured, and then isolated from the background with the “clear outside” option. The central area of the lesion, containing parasite or necrotic material, was selected by the freehand tool, measured, and deleted by the “cut” function to avoid erroneous measurements of color values similar to the collagen area. After that, “color threshold” tool settings were adjusted to identify color values corresponding to collagen, and the obtained area was selected and measured. The color threshold measurement was repeated three times, and the final collagen area was calculated as the mean of the three consecutive measurements and expressed as a percentage of the total area of the granuloma.

Evaluation of cytokeratin and vimentin expression in granulomas was accomplished using ImageJ “Color threshold”, as in the case with collagen. Different from the previous measurement, the color threshold system measured the area free from IHC staining, which was obtained by subtraction from the total area of the granuloma and the remaining measured area. The area positive to cytokeratin or vimentin was then calculated as the mean of three consecutive measurements and expressed as a percentage of the total area of the granuloma.

Mast cells and rodlet cells were manually counted using the ImageJ “Multi-point” tool on microphotographs of each granuloma taken at 400× magnification on slides stained with Giemsa and Masson’s trichrome, respectively, and their absolute number was registered. All data obtained from measurements achieved with the ImageJ software were recorded in a database.

### 2.6. Statistical Analysis

Microscopic features of the lesions were analyzed using Stata 11.2 software (StataCorp LP, College Station, TX, USA). Immunohistochemical results were compared using the non-parametric Kruskal–Wallis test with Dunn’s post hoc comparison. The chi-square test (χ^2^) was used to correlate the prevalence of parasites in examined organs. Furthermore, categorical and ordinal variables were compared using the Spearman rho (ρ) rank correlation coefficient. A value of ρ approximately equal to 1 indicates a good correlation, a value near 0 indicates a poor correlation, and a negative value indicates an inverse correlation. A *p*-value < 0.05 was considered significant.

## 3. Results

### 3.1. Parasitological Findings

**Figure 1:**
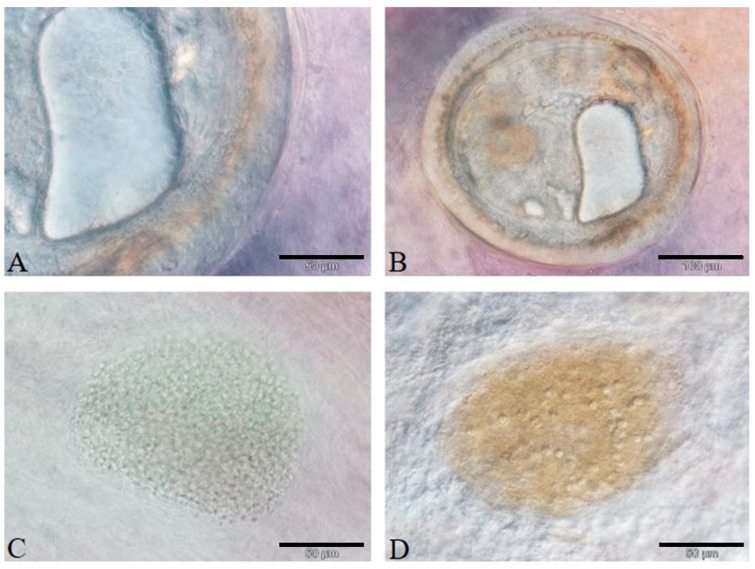
(**A**–**D**): Fresh smears of the parasites found in the examined organs of mugilids. (**A**): Detail of the circumoral spines of *Ascocotyle* (Phagycola) sp. metacercaria in the heart (bar 50 µm). (**B**): Encysted metacercaria in the heart (bar 100 µm). (**C**): Cyst of *Myxobolus* sp. faintly visualized in the heart of fresh specimens (bar 50 µm). (**D**): Spores in a melanomacrophage aggregate in the liver (bar 50 µm).

The microscopical examination of the fresh mounts of parasites allowed one to ascribe the TM to the Heterophyidae genera *Heterophyes*, *Stictodora*, and *Phagicola* (Ascocotyle) (Figure 1A,B) and the MP to *Myxobolus* sp. (Myxobolidae) (Figure 1C,D) in the liver, spleen, heart, and kidney (Figure 1).

### 3.2. Bacteriological Findings

The microbiological examination of all samples was negative for the presence of pathogenic bacteria and mycobacteria.

### 3.3. Histology and Immunohistochemistry Evaluation

Among the 239 sampled fish, 172/239 (71.96%) presented at least 1 parasite, of which 78/239 (32.63%) were affected only by TM and 40/239 (16.73%) only by MP, whereas in 54/239 fish (22.59%), both classes of parasites were detected. Concerning organ distribution, TM were detected in all examined organs, with a higher prevalence in the kidney (19.6%) and heart (14.2%), whereas MP were more frequently detected in the kidney (38.1%) and spleen (18.8%). Most of the fish affected by a single class of parasite presented an infection confined to a single organ (22.1% for metacercariae and 14.6% for Myxosporea).

*Mugil cephalus* showed a higher prevalence of TM in the heart (54.5%) and in the kidney for MP (36.3%) but without any significant statistical association. In *C. ramada*, TM were more frequent in the heart (12.7%), whereas MP mostly affected the kidney (57.2%, *p* < 0.05). The kidney was the most affected organ in *C. auratus* for both parasite classes (31.3% and 20.4% for metacercariae and Myxosporea, respectively, *p* < 0.05). In *Chelon labrosus,* MP were mostly found in the spleen (31.4%, *p* < 0.05). Details on the prevalence of organs affected by granulomas induced by both groups of parasites are shown in Table 2.

Most of the fish affected by a single class of parasite presented lesions confined to a single organ (35.9% for metacercariae and 40.5% for Myxosporea). Details on the percentages of each fish species affected by each of the parasite classes in one, two, three, or four evaluated organs are shown in Table 3.

Ziehl-Neelsen staining was negative for mycobacteria in all examined organs. The microscopic examination of the organs revealed a multifocal, mild-to-severe granulomatous inflammation around the parasites and three different stages of lesions. Out of 75 lesions, 25 were categorized as stage I, 27 as stage II, and 23 as stage III. Most of the selected fish showed the presence of only one stage of granuloma (68.1%), whereas 23.4% showed the presence of two stages, and in 8.5%, all stages were detectable.

**Figure 2:**
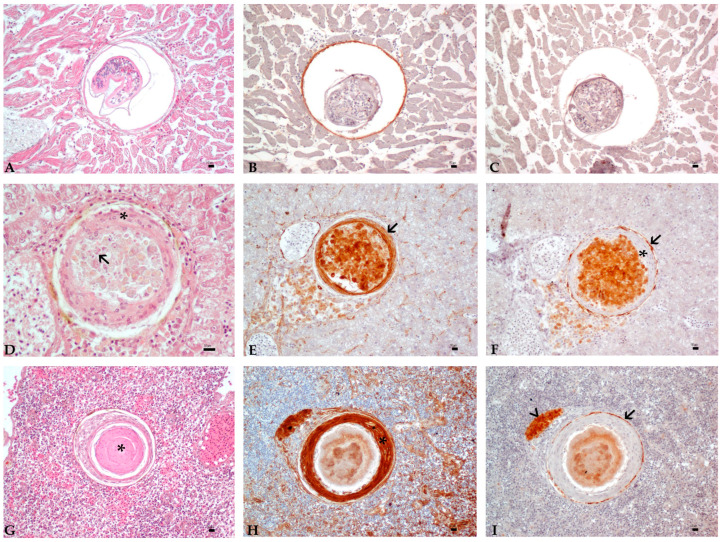
(**A**–**I**): Histology and immunohistochemistry panel of granuloma developmental stages in Mugilidae infected organs. Bar 10 µm. Serial section of the same samples in rows (**A**–C), (D–F) and (**G**–**I**). (**A**–**C**): Stage I (pre-granuloma stage). Heart. (**A**): TM encysted surrounded by a thin eosinophilic layer of compressed myocardial cells (HE, 20×). (**B**): IHC—positive labeling for the anti-cytokeratin antibody is expressed by parasite capsule (CKAE1/AE3 antibody, 20×). (**C**): IHC—absence of immunoreactivity to anti-vimentin antibody (Vimentin antibody, 20×). (**D**–**F**): Stage II (intermediate-stage granuloma). Liver. (**D**): Myxozoan spores (arrow) intermingled with necrosis and surrounded by a few layers of epithelioid cells (asterisk) (HE, 40×). (**E**): IHC—immunoreactivity for the anti-cytokeratin antibody in the EC layers (arrow) (CKAE1/AE3 antibody, 20×). (**F**): IHC—mild signal of immunoreactivity to anti-vimentin antibody in the fibroblast layer (arrow) around negative epithelioid cells (asterisk) in this stage (Vimentin antibody, 20×). (**G**–**I**): Stage III (late-stage granuloma). Spleen. (**G**): Central necrotic core (asterisk). (**H**): IHC—positive labeling for anti-cytokeratin antibody in a thicker layer of ECs in this stage (asterisk) (CKAE1/AE3 antibody, 20×). (**I**): IHC—positive labeling to the anti-vimentin antibody of the outer layer of fibroblasts (arrow). An aggregate of macrophages is occasionally observed in proximity to the lesion (arrowhead) (Vimentin antibody, 20×).

Stage I was termed as pre-granuloma stage and characterized by an intact parasite surrounded by a thin cyst wall, in association with scarce inflammatory cells. At this stage, positive labeling for anti-cytokeratin antibody was occasionally expressed by the parasite capsule, whereas immunoreactivity to anti-vimentin antibody was negative (Figure 2A–C).

Stage II was termed as intermediate-stage granuloma and characterized by parasites with loss of somatic details or partially destroyed parasites, but that were still recognizable in the center of the lesion, and often intermingled with a scarce to moderate amount of granular eosinophilic material and nuclear debris (necrosis). Outer encircling layers of flattened cells with a moderate to abundant eosinophilic cytoplasm, an ovoid vesicular nucleus with prominent, basophilic, and central nucleolus were detected around the degenerated parasite and necrosis. Due to these morphological features and to their strong and diffuse positive cytoplasmic signal to anti-cytokeratin CKAE1-AE3 antibody, these flattened cells were identified as epithelioid cells (Figure 2D–F). Moreover, an external layer composed of fusiform cells with scarce eosinophilic cytoplasm, an ovoid-to-elongated eccentric nucleus with dispersed chromatin, and 1–2 basophilic nucleoli, identified as fibroblasts, was sometimes detected loosely arranged around ECs. A scarce amount of collagen was occasionally found in this stage, intermingled with fibroblasts.

Stage III was designated as late-stage granuloma and characterized by a central eosinophilic core of necrosis, with parasite remnants only occasionally visible, and increased layers of epithelioid cells. The external wall was constituted by increased layers of fibroblasts strongly positive to anti-vimentin antibody (Figure 2G–I).

Scattered macrophages were detected near parasite cyst walls in stage I lesions and/or intermingled with necrotic material or scattered in the surrounding parenchyma in later stages. Aggregates of macrophages, frequently showing pigment-laden cytoplasm, were observed in association with the external layers of granulomas in 25/75 granulomas (Figure 2H,I).

**Figure 3:**
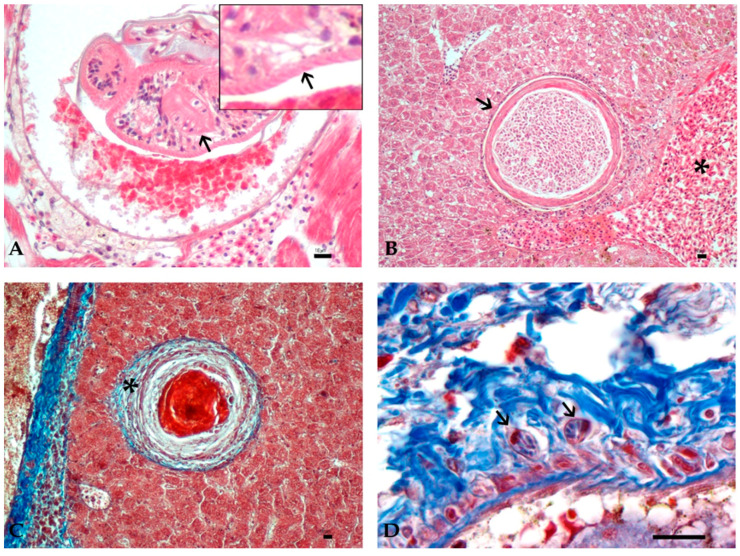
(**A**–**D**): Histological features of granuloma structure and cellular components in Mugilidae infected organs by histozoic parasites. Bar 10 µm. (**A**): Encysted metacercaria in the heart with evident ventral sucker (arrow). Inset. Magnification of cuticle spines (arrow) on larva surface (HE, 40×). (**B**): Plasmodium containing spores of myxozoan parasites surrounded by an eosinophilic capsule (arrow) in the liver close to a blood vessel (asterisk) (HE, 20×). (**C**): Collagen fibers in the outer layer of late-stage granuloma (asterisk) (MT, 20×). (**D**): Rodlet cells (arrows) in perivascular collagen (MT, 100×).

At histological examination, parasites were described based on characteristic features of the parasite classes, clearly discernible at stage I. Trematode metacercariae were detected in all examined organs, ranging from 150 to 400 microns in diameter, without body cavity and surrounded by a light eosinophilic capsule. Oral and ventral suckers, identifiable by the presence of a radially arranged musculature, were often recognizable, as well as a cuticle with a thickness of 2–4 microns with occasionally detectable cuticle spines that appeared as distinct eosinophilic linear structures (Figure 3A).

Histozoic plasmodia of myxozoans were found encysted in the parenchyma of the heart, liver, spleen, and kidney. Plasmodia, containing a variable number (until hundreds) of mature spores, were round to oval, ranging from 100 to 450 μm, and were delimited by a thin eosinophilic wall (Figure 3B).

In granulomas, collagen fibers were recognizable as blue filaments with Masson’s trichrome staining in the external layer of intermediate- and late-stage granulomas (Figure 3C).

**Figure 4:**
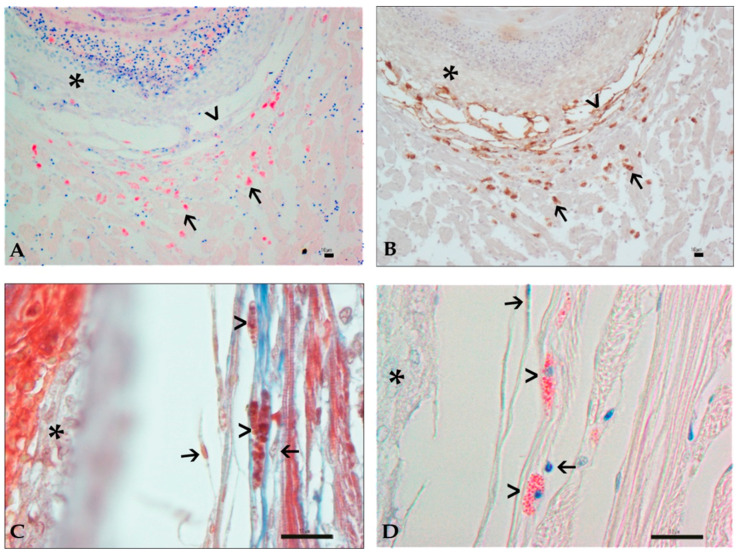
(**A**–**D**): Histological features of granuloma structure and cellular components in Mugilidae infected organs by histozoic parasites. Bar 10 µm. (**A**): Mast cells (arrows) near the fibroblast layer (arrowhead) and the EC layer (asterisk) of a late-stage granuloma (Giemsa, 20×). (**B**): Same detail of figure (**A**). Positive labeling to the anti-vimentin antibody in MCs (arrows) and fibroblast layer (arrowhead). The signal is absent in the EC layer (asterisk) (IHC, 20×). (**C**): Mast cells (arrowheads) in the external layer of late-stage granuloma in contact with fibroblasts (arrows) and blue collagen fibers. Epithelioid cell layer is visible (asterisk) (MT, Oil 100×). (**D**): Same detail of figure (**C**). Mast cells with evidenced granules (arrowheads) in contact with fibroblasts (arrows) and collagen fibers. Epithelioid cell layer is visible (asterisk) (Giemsa, Oil 100×).

Mast cells (MCs) appeared as ovoid cells with distinct borders and abundant cytoplasm containing numerous eosinophilic granules, brightly evidenced by Giemsa staining, and a central nucleus (Figure 4A).

Mast cells also showed a strong cytoplasmic positive signal to anti-vimentin antibody (Figure 4B). In both affected and unaffected samples, MCs were occasionally observed near vascular structures. In the pre-granuloma stage, MCs were close to parasites, whereas in the intermediate- and late-stage granulomas, MCs migrated to the more external layers. They were indeed more commonly observed intermingled with fibroblasts and sometimes detected in close contact to the fibroblasts membrane, elongated in shape with loosely arranged granules (Figure 4C,D).

Rodlet cells were observed in scarce numbers in all stages, mainly disposed near the parasite capsule or in the surrounding parenchyma. They were also observed near the main vascular structure both in parasitized and healthy samples (Figure 3D).

**Figure 5:**
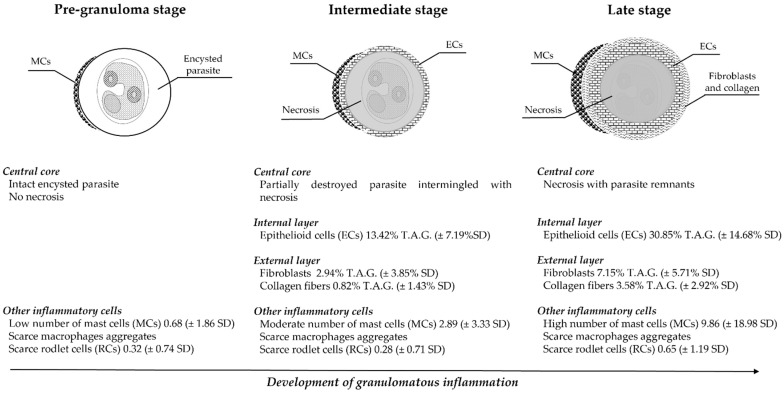
Stages and histological associated patterns of histozoic parasite granuloma formation in Mugilidae. SD = standard deviation; %T.A.G.= mean percentage of the total area of the granuloma.

The results of the structural components of granulomas and associated immune cells, as well as the description of histological parasite characteristics, were combined in a general scheme of possible developmental stages of granuloma formation (Figure 5).

### 3.4. Image and Statistical Analysis

Epithelioid cells showed a statistically significant positive correlation with granuloma progressive stages, showing an increase in parallel to the host immune response progression (Kruskal–Wallis χ^2^ (corrected for ties) = 62.686, *p* = 0.00010; Dunn’s post hoc test, pre-granuloma vs. intermediate *p* = 0.000002; pre-granuloma vs. late *p* = 0.000000; intermediate vs. late *p* = 0.000450). In the intermediate stage, ECs covered a mean area of 13.42% (± 7.19% SD) of the total area of the granuloma, whereas in the late stage, they reached approximately 1/3 of the total area of the granuloma (mean area 30.85% ± 14.68% SD).

The fibroblast layer increased with granuloma progression (Kruskal–Wallis χ^2^ (corrected for ties) = 38.963, *p* = 0.00010; Dunn’s post hoc test, pre-granuloma vs. intermediate *p* = 0.000842; pre-granuloma vs. late *p*= 0.000000, intermediate vs. late *p* = 0.002460). Fibroblasts covered averagely 2.94% (± 3.85% SD) of the total area of the granuloma in stage II, reaching a mean area of 7.15% (± 5.71% SD) in the late stage.

Statistical analysis evidenced that collagen had a significant increase in intermediate- and late-stage granulomas (Kruskal–Wallis χ^2^ (corrected for ties) = 36.791, *p* = 0.00010) (Dunn’s post hoc test, pre-granuloma vs. late *p*= 0.00000; intermediate vs. late *p* = 0.000231). Moreover, collagen showed a statistically significant association with the fibroblast layer in the intermediate-stage granuloma (ρ =0.6257 *p* < 0.05). The collagen layer covered a mean area of 0.82% (± 1.43% SD) in the intermediate stage and a mean area of 3.58% (± 2.92% SD) in the late-stage granuloma.

Mast cells were observed in all stages of the lesion development, showing an increase from the pre-granuloma stage, with a mean number of 0.68 (± 1.86 SD), to the intermediate- (2.89 ± 3.33 SD) and late-stage granulomas (9.86 ± 18.98 SD). Statistical analysis of MCs revealed a significant increase in number in the pre-granuloma and intermediate-stage granuloma (Kruskal–Wallis χ^2^ (corrected for ties) =29.531, *p* = 0.00010 (Dunn’s post hoc test, pre-granuloma vs. late *p* = 0.00000; pre-granuloma vs. intermediate *p* = 0.000773). Moreover, a significant positive correlation between the collagen area and the number of mast cells was observed (ρ =0.2701, *p* < 0.05). Evaluation of the association between the number of mast cells and the granuloma proximity to vascular structures revealed no significant statistical association.

Rodlet cells were detected with a mean number of 0.32 (± 0.74 SD) in the pre-granuloma stage, 0.28 (± 0.71 SD) in the intermediate stage, to 0.65 (± 1.19 SD) in the late-stage granuloma. Moreover, conversely to MCs, no statistically significant difference in the number of the RCs was observed in the different stages of host immune response.

Macrophages aggregates were found associated in 16% of the pre-granuloma stage, 44.4% in the intermediate stage, and 39.1% in the late-stage granuloma. However, no statistically significant variation was observed between their presence and the different stages of granulomas.

Quantification of granuloma’s proximity to vascular structures gave the following results: 33.3% of lesions were considered in the “close” category, 33.3% fell in the “near” category, and 33.3% in the “far” category. The association between the distance of granulomas from vessels and granuloma stages was not statistically significant (*p* > 0.05).

## 4. Discussion

Inflammatory lesions associated with TM and MP were detected in (71.9%) of the examined fish, confirming the observation of other authors that reported Mugilidae as a widely parasitized species [38]. The parasitological examination of grey mullets allowed one to identify three genera of TM: *Heterophyes* sp., *Stictodora* sp., and *Phagicola* sp., belonging to the heterophyid family (Trematoda: Heterophyidae). Heterophyids, which have a fish-eating bird or mammals as the definitive host, are widely encountered parasites of mullets in Sardinian brackish waters [51,59,60] as well as in other Mediterranean areas [38,42,48]. Pre-granulomas and granulomas associated with metacercariae were detected in all examined organs, with a higher prevalence in the kidney and heart. TM have a wide range of tissue distribution, as they have been reported in almost every organ of mugilids [60,61,62,63,64,65].

Myxozoan parasites, especially the Myxobolidae family, have been extensively described in mugilids worldwide [50,66,67,68,69]. In the present study, myxozoan plasmodia were detected in all organs, with a higher prevalence in the kidney and spleen. These data are aligned with the literature of *Myxobolus* spp. infection in many Mugilidae species from the Mediterranean area, which described encysted plasmodia in different tissues [70,71,72,73,74,75]. Furthermore, albeit the pre-granulomas and granulomas were detected in all examined organs, most fish showed lesions associated with TM and MP only in one organ. This result could reflect the preference of a parasite species for a specific organ, as reported for some species of myxozoan parasites [76].

Parasite infection in fish can be the result of a single contact with intermediate hosts or can be due to multiple exposures to parasites, leading to the formation of younger parasite cysts near to older and almost resolved infection [43]. The granuloma stages distribution in the selected samples reflected these observations and, whereas most of the fish presented a single granuloma stage, probably as a result of a single contact with intermediate hosts, one-third of them resulted in detection of multiple granuloma stages, thus suggesting that these fish were likely subjected to prolonged exposure to the intermediate hosts.

The response of the fish immune system after parasite invasion is variable and reliant on a multitude of factors (fish age, infected organ, class of parasite), with a different outcome in terms of damage extent to host tissues [24,77,78].

In the pre-granuloma stage (stage I), parasites were detected encysted in the parenchyma of several organs, generally surrounded by a compact eosinophilic layer, composed of compressed parenchymal cells because of the cyst pressure. The inflammatory response in this phase was absent or mild, and when present, mainly characterized by a limited number of mast cells close to the parasite wall or scattered in the surrounding parenchyma, and by occasional macrophages. This feature has been reported by other authors in early encysted TM [43,64] and histozoic forms of MP [79]. The paucity of the immune response in this early phase could be related to the ability of the parasite to escape the host immune system or to regulate the mounting inflammatory process, as reported for trematode metacercariae and *Myxobolus* spp., respectively [78].

The hallmark of the intermediate-stage granuloma (stage II) was the presence of epithelioid cells that encircled the parasites. These cells were discernible with IHC staining by the strong positive labeling to anti-cytokeratin antibody (CKAE1/AE3), as was reported by studies in other fish species [2,28]. The intermediate stage represented the host immune system response directed toward parasite isolation. Encircling and sequestration of parasites by a layer of epithelioid cells were observed in granulomas belonging to both groups of parasites, and it is also a widely reported feature in the course of tissue-dwelling parasite infection [2,80,81,82,83]. It is well recognized that the host immune response against parasites differs depending on the location where the infection takes place. In the course of parasitism of luminal organs, such as the intestine, immune cells expel parasites by the local release of vesicle content at the site of parasite infection and by the stimulation of synergist mechanisms (increase in mucous production, stimulation of peristalsis, etc.) [84,85,86,87]. On the contrary, in parenchymal organs where a proper elimination of the parasite is not achievable, encapsulation is the mechanism employed to limit parasite spread [10,11,37,88].

Lastly, late-stage granuloma (stage III) represented the late phase of chronic inflammation with the highest quantity of immune cells, which led to the resolution of parasite infection. In this stage, due to the presence of central necrosis and the lack of parasite remnants, the results of Ziehl-Neelsen staining excluded the presence of mycobacterial infection.

Epithelioid cells and fibroblasts, which are recently considered strictly associated with immune response, were found in the largest amount in the late-stage granuloma. Fibroblasts were more prominent and evidenced by a diffuse membrane immunoreactivity to anti-vimentin antibody, a marker for the intermediate filament of mammalian mesenchymal cells that is preserved in some fish species [89]. Fibroblasts have been frequently reported in association with parasite granulomas in fish tissues, variably intermingled with collagen fibers, to further prevent the spread of parasites [10,30,90,91]. This has been confirmed in the present study since fibroblasts acted to delimitate granuloma expansion and contributed to tissue remodeling by increased production of collagen, especially in intermediate- and late-stage granulomas. In particular, the strong statistical correlation found between fibroblasts and collagen fibers limitedly to intermediate-stage granuloma (*p* < 0.001) could be explained by the fact that in this phase, both fibroblasts and collagen were present in similar proportions. However, in stage III, even if both categories were numerically increased, collagen production augmented fourfold compared to the twofold rise in fibroblasts. The biological explanation for this difference could be that in late stages, fibroblasts have accomplished their role and their number start to decrease, whereas collagen, which represents fibroblast’s final product, is at the highest levels. There is evidence that fibroblasts, although not proper immune cells, make an important contribution in the host response against parasites, by crosstalk with other immune cells, as demonstrated in mammals and fish species [32,92]. An interesting link between teleostean MCs and fibroblasts has been recently evidenced, particularly addressing the role of MCs in promoting fibroblast collagen production [11,33]. Mast cell function in the pathogenesis of human fibrosis has been extensively investigated and different mechanisms of their action on fibroblasts have been defined, such as the piecemeal release of cytoplasmic vesicles in the interstitium or via the transfer of molecules by direct cell-to-cell contact (transgranulation) [34,93]. This finding is in accordance with what was reported by other authors on the close association of MCs and fibroblasts in parasite granulomas in teleosts [33].

In this work, microscopic examination of stages II and III granuloma revealed the presence of mast cells grouped around the external layer, adherent to fibroblasts, thus leading to the hypothesis that a transgranulation mechanism of MCs towards fibroblasts could be plausible. The statistically significant association of MCs with the collagen layer (ρ = 0.2701, *p* < 0.05) reinforced the hypothesis that action of MCs could be more specifically addressed to the regulation of fibroblasts in collagen production, rather than to fibroblast recruitment in the site of infection, in intermediate- and late-stage lesions.

The results of the present work suggest that the role of mast cells as promoters of collagen production may not be the only mechanism in which MCs are involved against histozoic parasites. Indeed, the low number of MCs observed in the pre-granuloma stage and their distribution around the parasite capsule suggest that their action could be involved, synergistically with macrophages, in promoting host response, perhaps by the recruitment of other immune cells at the site of infection. This mechanism, recognized in mammalian mast cells during parasite infection, has been supposed in teleosts, where MCs are recognized as first effectors during parasitism [83]. Mast cell migration from blood vessels to the site of infection has been observed in fish species [94]. In addition to a circulating population, the existence of a local reserve of MCs, which could proliferate in case of parasitism, has been confirmed in mammals and supposed in fish species [11,95]. The latter situation likely represents the present case, where the lack of statistical association between mast cell number and the proximity to blood vessels in the various developmental stages suggests that the number of mast cells associated with granulomas could be the result of the proliferation of a resident population, rather than the consequence of migration from the peripheral bloodstream. This finding is in accordance with what was reported by Dezfuli et al. (2017) [11], which showed the positivity to the proliferating cell nuclear antigen (PCNA) in mast cells of the liver of *Gymnotus inaequilabiatus* (Valenciennes, 1839) infected by nematode larvae [11].

Another group of immune cells, known as rodlet cells, that represent a unique element of the teleostean immune system [36,96], was found in the present work in a low number. Furthermore, the absence of a recognizable organization around parasite lesions and the lack of any significance at statistical analysis suggested that RCs’ role against histozoic parasites could be less specific and maybe mostly committed to cooperation with other immune cells in the host inflammatory response, rather than a direct action on the parasite, as described in parasite infection of the gills and intestine [97,98].

Macrophage aggregates were inconsistently found at the periphery of the granulomas, sometimes containing brownish cytoplasmic pigments. Aggregates of pigment-laden macrophages (MMAs) are normally present in the liver and spleen of many teleosts and can vary in number and dimension under the influence of several factors, such as age, stress, diseases, and pollution [99]. MMAs have also been reported in the liver, spleen, kidney, and heart around granulomas induced by larvae of helminths [10,11,37]. In the present study, the presence of aggregates of macrophages associated with the granuloma showed a variable presence not statistically correlated with granuloma developmental stages. This result suggests that their presence could be perhaps influenced by other factors, such as age and fish species, as reported by other authors on studies on exposure to pollutants, diet, environmental stress, and fish aging [99,100].

The present study underlined that host immune response against the two groups of parasites revealed a substantial superimposability, leading to a common model of development of the immune response against trematode metacercariae and myxozoan parasites.

## 5. Conclusions

Parasitic disease is a relevant issue for fish health and represents a challenge between the attempt of parasites to survive and the host’s action aimed to prevent the spread of infection.

This study, by combining histopathology with immunohistochemical methods, represents an effort to expand the knowledge of inflammatory reactions associated with parasites in fish organs and their staging. This makes it conceivable that the identified histological patterns could be reliable tools to staging the granulomatous response associated with histozoic parasites and represent an effort to classify granulomas of different origins.

Further analyses should be conducted to understand mechanisms of granuloma formation induced by different species of parasites in various experimental models, as well as the development of early detection methods, especially in species of great commercial interest for human consumption.

## Figures and Tables

**Table 1:** Fish species collected during summer and autumn 2014.

	Summer Sampling				Autumn Sampling				
	CH	ST	MI	MA	CH	ST	MI	MA	Total
*Mugil cephalus*	0	6	0	0	0	5	0	0	11
*Chelon labrosus*	16	10	0	0	0	1	0	8	35
*Chelon auratus*	1	13	0	29	0	23	0	17	83
*Chelon ramada*	13	1	30	0	30	1	30	5	110
Total	30	30	30	29	30	30	30	30	239

Key: CH = Calich lagoon, ST = San Teodoro lagoon, MI = Mistras lagoon, MA = Marceddì lagoon.

**Table 2:** Prevalence of organs affected by lesions associated with two parasite classes in different Mugilidae species.

	TM				MP			
	1	2	3	4	1	2	3	4
*Mugil cephalus*	36.36% (4/11)	36.36% (4/11)	9.09% (1/11)	9.09% (1/11)	54.54% (6/11)	0	0	0
*Chelon labrosus*	45.71% (16/35)	5.71% (2/35)	0	0	17.14% (6/35)	8.57% (3/35)	5.71% (2/35)	0
*Chelon auratus*	45.78% (38/83)	20.48% (17/83)	2.40% (2/83)	1.20% (1/83)	43.37% (36/83)	7.22% (6/83)	1.20% (1/83)	0
*Chelon ramada*	25.45% (28/110)	18.18% (20/110)	2.72% (3/110)	0	44.54% (49/110)	12.72% (14/110)	0	0
Total	35.98% (86/239)	17.99% (43/239)	2.51% (6/239)	0.83% (2/239)	40.58% (97/239)	9.62% (23/239)	1.25% (3/239)	0

Key: = TM = trematode metacercariae; MP = myxozoan plasmodia. 1 = on one organ involved; 2 = two organs involved; 3 = three organs involved; 4 = four organs involved.

**Table 3:** Percentages of each Mugilidae species affected by each of the parasite classes in one, two, three, or four evaluated organs.

	Heart		Liver		Spleen		Kidney		*p* Value	
	TM	MP	TM	MP	TM	MP	TM	MP	TM	MP
*Mugil cephalus*	6/11 (54.5%)	-	4/11 (36.3%)	3/11 (27.2%)	4/11 (36.3%)	3/11 (27.2%)	5/11 (45.4%)	4/11 (36.3%)	*p >* 0.05	*p >* 0.05
*Chelon labrosus*	2/35 (5.7%)	1/35 (2.8%)	5/35 (14.2%)	8/35 (22.8%)	2/35 (5.7%)	11/35 (31.4%)	4/35 (11.4%)	7/35 (20%)	*p >* 0.05	*p* < 0.05
*Chelon auratus*	12/83 (14.4%)	-	10/83 (12%)	16/83 (19.2%)	2/83 (2.4%)	14/83 (16.8%)	26/83 (31.3%)	17/83 (20.4%)	*p* < 0.05	*p* < 0.05
*Chelon ramada*	14/110 (12.7%)	2/110 (1.8%)	9/110 (8.1%)	13/110 (11.8%)	5/110 (4.5%)	17/110 (15.4%)	12/110 (10.9%)	63/110 (57.2%)	*p >* 0.05	*p* < 0.05
Total	34/239 (14.2%)	3/239 (1.2%)	28/239 (11.7%)	40/239 (16.7%)	13/239 (5.4%)	45/239 (18.8%)	47/239 (19.6%)	91/239 (38.1%)		

Key: = TM = trematode metacercariae; MP = myxozoan plasmodia. Chi-square test (χ^2^) was used to correlate the prevalence of parasites in each examined organ.

## Data Availability

The data presented in this study are available upon request from the corresponding author.
